# Health Care Use among Medicare Beneficiaries with HIV and Depression during the COVID-19 Pandemic—United States, 2020

**DOI:** 10.3390/healthcare11081126

**Published:** 2023-04-13

**Authors:** Man-Huei Chang, Ramal Moonesinghe, Benedict I Truman

**Affiliations:** 1 National Center for HIV, Viral Hepatitis, STD, and TB Prevention, Centers for Disease Control and Prevention, Atlanta, GA 30329, USA; 2 Office of Genomics and Precision Public Health, Centers for Disease Control and Prevention, Atlanta, GA 30345, USA

**Keywords:** HIV, depression, facilities and services utilization, Medicare, COVID-19

## Abstract

Access and use of health care services are essential to health and well-being for people with HIV and HIV-related comorbidities. Health care use during the COVID-19 pandemic among Medicare beneficiaries (MBs) with concurrent HIV and depression has not been investigated. We used 2020 Medicare data to assess the percentage of MBs with claims for HIV and depression who also received hospitalization, outpatient diagnostic services, drug treatment, and outpatient procedures. We assessed person-level association between service receipt and HIV and depression, adjusting for known risk factors. MBs with claims for HIV and depression were more likely than those with neither claim to have claims for short-stay hospitalization, long-stay hospitalization, outpatient diagnostic services, prescription drugs, or outpatient procedures, supplies, and products. Non-White beneficiaries were more likely than White beneficiaries to be hospitalized but were less likely to receive drug treatment, outpatient diagnostic services, or outpatient procedures, supplies, and products during the pandemic. Significant disparities in health care use by race/ethnicity existed among MBs. Policymakers and practitioners can use these findings to implement public health policies and programs that reduce disparities in health care access and optimize use among vulnerable populations during a public health emergency.

## 1. Introduction

Health care utilization, delivery, and access were essential to improving the general health and well-being of populations during the COVID-19 pandemic [1]. This is particularly true for people with HIV and HIV-related comorbidities. Consistent access to HIV health care, including testing, antiretroviral therapy (ART) prescriptions, ART adherence, and behavioral and mental health support, is critical to positive health outcomes. An individual’s health status is a major determinant of health care utilization; however, social and biological factors within groups of people, such as poverty, geographic area of residence, education, race and ethnicity, sex, age, disability status, economic stability, community safety, and availability of adequate housing, also have an impact on health care utilization [2]. Research on health care utilization can identify opportunities for policymakers and practitioners to implement public health policies and programs that reduce disparities in health care access and optimize use among vulnerable populations.

The COVID-19 pandemic disrupted access to and use of health care services in the United States [3,4]. New HIV infections reported annually decreased 17% from 36,940 infections diagnosed in 2019 to 30,635 diagnosed in 2020 in the United States [5]. The decrease was likely attributable to limited availability of health care services, hesitancy among people to access health care, and labor and material shortages for HIV testing during the COVID-19 pandemic [5]. Older people with HIV infection and other underlying health conditions who were not on antiretroviral treatment were more likely to become seriously ill if they got COVID-19 during 2020 [6]. People living with untreated HIV were disproportionately affected by mental disorders [7,8]. The prevalence of depression symptoms in the United States was more than 3-fold higher during COVID-19 compared with before the COVID-19 pandemic [9]. The Centers for Disease Control and Prevention (CDC) also reported that 42% of Americans reported symptoms of depression or anxiety in December 2020, compared with 11% in 2019 [10].

Significant increases in depression and anxiety among people with HIV were found due to the impact of COVID-19 pandemic during 2020 [11,12]. Javanbakht and colleagues found interruptions in mental health care were associated with substance abuse during the pandemic, suggesting substances such as cannabis were being used to manage depression and anxiety in the absence of access to mental health care [11]. The COVID-19 pandemic also led to significant decreases in HIV-related service utilization (e.g., access and use of HIV health care services, HIV testing and diagnoses, and ART initiation and refills) [3]; in contrast, mental health encounters increased among people with HIV infection [4].

Studies have shown that people in all age and demographic groups with social disadvantages and certain chronic conditions, including depression, HIV, and other causes of immune suppression, were at the highest risk of being hospitalized with COVID-19 during the pandemic; COVID-19 and chronic disease burden varied by demographic attributes [13]. CDC reported that African Americans accounted for 42% of the HIV infections in the United States in 2020 (www.cdc.gov/hiv/, accessed on 11 April 2023). Racial and ethnic disparities in HIV incidence, prevalence, testing, and receipt of preventive health care have been well-documented, along with the variation of those disparities with age, sex, sexual orientation. However, health care use during the COVID-19 pandemic among Medicare beneficiaries with concurrent HIV and depression has not been investigated. The association of health services utilization among MBs with both HIV and depression claims by race and ethnicity has not been estimated yet.

Medicare beneficiaries (MBs) include adults aged ≥65 years and younger people who have end-stage renal disease or amyotrophic lateral sclerosis (Lou Gehrig’s disease) or who have a disability that qualifies them to receive Medicare benefits below age 65 years (www.medicare.gov/, accessed on 11 April 2023). MBs are U.S. residents who are qualified to receive medical and social services paid for by the Medicare insurance program operated by the U.S. government since 1965. Some MBs are at high risk for HIV infection because of substance use and also have comorbid conditions related to HIV, such as immune suppression and clinical depression. Thus, Medicare claims data offer an opportunity to examine the association of health care services utilization among a diverse population with both HIV and depression. This study aimed to describe attributes of MBs with claims for HIV and depression and estimate health care services utilization among MBs with fee-for-service claims with or without HIV and depression during COVID-19 pandemic in 2020. We hypothesized that during the COVID-19 pandemic in 2020 (1) health care services utilization was greater among MBs with HIV and depression than among those without those conditions; and (2) greater utilization among MBs was associated with concurrent HIV and depression, independent of age, sex, race/ethnicity, U.S. region, place of residence, comorbidity, and hospital emergency room visits.

## 2. Materials and Methods

### 2.1. Data and Study Outcomes

We used 100% Medicare fee-for-service claims data collected during 2020; data were accessed through the Centers for Medicare and Medicaid Services (CMS; https://www.cms.gov/, accessed on 11 April 2023) Virtual Research Data Center. We used CMS predefined flags to identify MBs who had claims for HIV infection and depression. Detailed definitions of HIV and depression are available in the Chronic Conditions Data Warehouse [14].

Beneficiaries were classified as having both HIV and depression claims, HIV claims and no depression claims, depression claims and no HIV claims, or neither HIV nor depression claims. Five health care services utilization outcome variables included: (1) short-stay hospitalization, (2) long-stay hospitalization, (3) outpatient diagnostic services, (4) drug treatment services, and (5) outpatient procedures. Short-stay hospitalization and long-stay hospitalization were classified using the indicator variable of inpatient hospital and facility stays covered in MedPAR [15,16]. Swing-bed hospital designation for short-term hospitals and swing-bed designation for critical access hospitals were also included in short-stay hospitalization. Stays in skilled nursing facilities were excluded from the analysis. Outpatient diagnostic services were defined as an MB with claims for receiving care from outpatient providers with *International Classification of Diseases, Tenth Revision* (www.cdc.gov/nchs/icd/icd10.htm, accessed on 11 April 2023) codes for any conditions. Drug treatment services were defined as an MB with Part D coverage who was treated with drugs and filed a prescription drug claim to CMS using a National Drug Code for any condition. Outpatient procedures were defined as an MB with claims for physician services and other health care services with the Current Procedural Terminology codes, or any procedures, supplies, products, or services provided with codes of the Healthcare Common Procedure Coding System (HCPCS). Each health care services utilization variable was coded as yes (≥1 service) or no (service not provided).

### 2.2. Study Covariates

We calculated frequencies of MBs with both HIV infection and depression claims by age group (≤64, 65–74, 75–84, or ≥85 years), sex (male or female), race/ethnicity (non-Hispanic White (NHW), non-Hispanic Black (NHB), Hispanic, Asian/Pacific Islander (A/PI), American Indian/Alaska Native (AI/AN), or other/unknown), U.S. census region of residence (Northeast, West, Midwest, or South), metropolitan or nonmetropolitan residence (metropolitan (large central metropolitan and large fringe metropolitan, medium metropolitan, or small metropolitan), or nonmetropolitan (micropolitan and noncore)) [17], Medicare status code (aged without end-stage renal disease (ESRD), aged with ESRD, disabled without ESRD, disabled with ESRD, ESRD only, or not enrolled in Medicare A or B).

For the comorbidity variable, we included 20 selected conditions among MBs with HIV and depression as follows: acute myocardial infarction, atrial fibrillation, chronic kidney disease, chronic obstructive pulmonary disease, diabetes, heart failure, hyperlipidemia, hypertension, ischemic heart disease, stroke, colorectal cancer, lung cancer, urologic cancer, anxiety disorders, drug use disorder, leukemias and lymphomas, opioid use disorder, liver disease, peripheral vascular disease, and viral hepatitis. These conditions were selected because they are correlated with HIV or depression [18,19]. Comorbidities for each beneficiary were categorized as 0, 1–3, 4–5, or ≥6 conditions. ESRD was coded as yes or no based on the MB’s entitlements. We also included hospital outpatient emergency room visits (≥1 vs. 0) and calculated average length of hospital stay (0 days, 1 day, 2–5 days, 6–10 days, and ≥11 days).

### 2.3. Analysis

We computed percentages of MBs with HIV or depression claims during 2020, by demographic, geographic, and clinical factors. Next, we calculated the percentage of health care services among MBs with both HIV and depression in each demographic group. We used multivariable logistic regression to examine the association between concurrent HIV and depression and each of the five health care services utilizations, adjusting for demographic, geographic, and clinical risk factors. Lastly, we calculated adjusted odds ratios (aOR) for each health services utilization outcome, stratified by race/ethnicity. We used a 2-tailed chi-squared test for significance at *p* < 0.05. All analyses were performed by using SAS^®^ 9.4 and SAS Enterprise Guide^®^ 7.1 (SAS Institute, Inc., Cary, NC, USA) in the secured environment of the CMS Virtual Research Data Center through the Chronic Condition Warehouse [20]. The data used in this study were de-identified by CMS and were subject to a data-use agreement between CDC and CMS. As such, CDC deemed the study exempt from institutional review board review.

## 3. Results

### 3.1. Descriptive Characteristics

Table 1 presents the characteristics of MBs across the four categories of HIV and depression-related FFS claims during 2020. Of approximately 40.4 million beneficiaries with FFS claims in 2020, only 46,523 (0.11%) MBs had claims for both HIV and depression, 74,389 (0.18%) had claims for HIV but not depression, 6,542,197 (16.8%) had claims for depression only, and 33.8 million (83.2%) did not have claims for HIV or depression. MBs with both HIV and depression claims were more likely than those without HIV or depression claims to be aged ≤ 64 years old (68.6% vs. 11.0%), male (70.9% vs. 49.8%), NHB (34.9% vs. 9.3%), disabled (64.1% vs. 10.2%), residing in the Northeast or South region (66% vs. 55.5%), residing in a metropolitan area (91.1% vs. 80.2%), and to have ESRD (5.6% vs. 1.0%); they were also more likely to have ≥1 comorbid conditions (95.7% vs. 63.2%). Compared with MBs without claims for HIV or depression in 2020, those with claims for both HIV and depression were more likely to receive medical services requiring short-stay (34.5% vs. 10.8%) or long-stay (5.3% vs. 0.8%) hospitalizations, have longer hospital stays (35.9% vs.11% MBs stayed ≥1 days in a hospital), have ≥1 emergency room visits (53.3% vs. 18.1%), require outpatient diagnostic services (98.1% vs. 74.7%), fill a prescription drug (91.0% vs. 53.1%), and use outpatient procedures, supplies, and products (98.1% vs. 74.7%) for any condition.

### 3.2. Health Care Services Utilization

Table 2 presents health care services utilization among MBs with both HIV and depression claims by demographic, geographic, and clinical characteristics. Older MBs with both HIV and depression claims had more health care services claims for any condition than younger MBs. For example, the percentage of short-stay hospitalization for any condition was highest among MBs with HIV and depression claims at age ≥ 85 years (59.3%), followed by 45.2% at age 75–84 years, 35.8% at age 65–74 years, and 32.9% at age < 65 years. MBs with HIV and depression claims who were female, NHB, disabled with ESRD, resided in the South region, had ≥6 comorbid conditions, had ≥1 emergency room visits, and were more likely to have short-stay hospitalizations than other population subgroups. In general, similar demographic, geographic, and clinical patterns in service utilization among MBs with HIV and depression claims were also observed for long-stay hospitalization, outpatient diagnostic services, drug treatment services, and outpatient procedure services for any condition.

### 3.3. Association between Health Care Services Utilization and MB Characteristics and Claims

A significant association between service utilization for any condition and MBs with claims for both HIV and depression was observed after controlling for age, sex, race/ethnicity, region, metropolitan, comorbidity, and emergency room visits (Table 3). During 2020, MBs who had claims for both HIV and depression were more likely (aOR = 1.6; 95% CI = 1.5–1.6) to have claims for short-stay hospitalization for any condition than those who had neither HIV nor depression claims. MBs with depression claims but no HIV claims had 1.5 times the odds of short-stay hospitalizations (95% CI = 1.5–1.5), while MBs with HIV claims but no depression claims had similar odds of short-stay hospitalizations (95% CI = 1.0–1.1) as those without HIV or depression claims. Similarly, MBs with both HIV and depression were more likely to have claims for long-stay hospitalization (aOR = 2.5; 95% CI = 2.4–2.6), outpatient diagnostic services (aOR = 4.4; 95% CI = 3.8–4.5), drug treatment services (aOR = 4.9; 95% CI = 4.7–5.1), and outpatient procedure services (aOR = 4.2; 95% CI = 3.8–4.5) for any condition during 2020 than MBs with neither HIV nor depression claims. All five types of health care services utilization were also strongly associated with ≥1 comorbidities and ≥1 emergency room visits. Moreover, NHB, Hispanic, and AI/AN beneficiaries were more likely to be hospitalized and less likely to receive drug treatment services, outpatient diagnostic services, and outpatient procedure services than NHW beneficiaries during 2020.

### 3.4. Association of Health Care Services Utilization and MB Claims, by Race/Ethnicity

For NHW, NHB, and Hispanic MBs, we observed a statistically significant association between HIV and depression claims and each of the five types of health care services utilization after controlling for age, sex, region, metropolitan, comorbidity, and emergency room visits (Table 4); however, associations varied slightly by racial and ethnic group. Compared with those who did not have HIV or depression claims, NHW MBs with claims for both HIV and depression had approximately 1.6 times the odds, NHB MBs had 1.7 times the odds, and Hispanic MBs had 1.5 times the odds of short-stay hospitalization. Similarly, associations varied by race/ethnicity across the other health care services utilizations. For example, NHW, NHB, and Hispanic MBs with claims for both HIV and depression were 4–6 times (aOR = 4.2, 5.5, and 6.1, respectively) more likely to receive drug treatment services than those who did not have HIV or depression claims.

## 4. Discussion

MBs with both HIV and depression claims during 2020 were a small proportion of the overall MB population and had sociodemographic characteristics, geographic distributions, and clinical risk profiles that differed from MBs with neither HIV nor depression claims. MBs with claims for both HIV and depression were predominantly younger, male, non-Whites, disabled, resided in metropolitan areas, had ESRD, and had co-occurrence of chronic conditions. Significant differences in health care services utilization were observed for MBs with claims for both HIV and depression versus those with claims for neither HIV nor depression during 2020. Compared with those MBs without HIV or depression, MBs with claims for both HIV and depression were more likely to have short-stay or long-stay hospitalizations, longer hospital stays, require outpatient diagnostic services, be treated with prescription drugs, and require outpatient procedures, supplies, and products.

Prior studies have well demonstrated that people living with HIV are at increased risk for mental health disorders, mortality, poor quality of life, disease burden, and developing serious illness as a result of COVID-19 [21,22]. Our findings on factors affecting health care services utilization align with prior studies that found that people who are older, NHB or AI/AN, disabled with ESRD, have more comorbid conditions, and have both HIV and depression, were more likely to be hospitalized during the COVID-19 pandemic [13,23,24,25]. Our findings also demonstrated that MBs with claims for both HIV and depression required more medical services and treatment. In March 2022, the Department of Health and Human Services (DHHS) announced nearly USD 44 million to strengthen mental health and substance use services for populations at risk for or living with HIV/AIDS [26]. This funding increases support and treatment services for those underserved racial and ethnic monitory populations living with or at risk for HIV/AIDS. During the COVID-19 pandemic, DHHS interim guidelines recommended several critical steps to assist people with HIV in medical services, treatment, and health care. For example, drug providers (e.g., Medicaid, Medicare) waived restrictions on drug-supply quantity, increased virtual communication and care for counseling services, and expanded telehealth services [27]. Chang and colleagues reported that telehealth availability for Medicare beneficiaries increased substantially during the COVID-19 pandemic [28]. The expansion of telehealth coverage by Medicare [29] during the pandemic might have promoted access to virtual care services, and in part, contributed to the utilization of diagnostics services during the pandemic that we observed in this study.

Similar to other studies that found racial and ethnic health disparities related to COVID-19 [30,31], we observed an inequality in health services utilization among MBs with both HIV and depression claims by race and ethnicity; the associated effects varied slightly by different type of health service. Although some systematic reviews and meta-analyses failed to find evidence of higher risk for COVID-19 among people living with HIV [32,33], the majority of studies reported that COVID-19 had a negative impact on HIV and depression treatment, care, surveillance, and research, particularly among people residing in marginalized communities [4,21]. Our results indicate that non-White MBs (expect for Asian/Pacific Islander MBs) had slightly higher odds of hospitalization but received less outpatient diagnostic services, drug treatment, and outpatient procedure supplies, and products during the COVID-19 pandemic. This might suggest the existing and persistent racial and ethnic disparities in health care services among U.S. communities continued to pose significant challenges for public health interventions during the COVID-19 pandemic. Accordingly, eliminating health disparities is one of the overarching goals of *Healthy People* 2030 [34]. Addressing social determinants of health that contribute to health disparities, including structural racism or systemic bias, is essential to achieving health equity. For HIV care, continued efforts to provide timely and effective access to health care are needed to increase the proportion of people who receive HIV medical care to 95% by 2030 [35].

Our results also indicate that MBs with claims for depression only were more likely to be admitted to a short-stay or long-stay hospitalization during the COVID-19 pandemic. MBs with claims for HIV only had significantly higher odds of being treated with drugs, receiving outpatient diagnostic services, and using outpatient procedure services during the pandemic than those without HIV or depression claims. However, studies have reported that while the pandemic has led to an increase in depression among people living with HIV [11,36], it has also led to significant interruptions in outpatient visits for HIV care and mental health care [3,4]. Our results support the importance of integrating HIV and mental health services, treatment, and interventions as outlined in a recent report published by the Joint United Nations Programme on HIV/AIDS and the World Health Organization [37]. Increased risk for HIV infection and mental health conditions is associated with lower retention in HIV health care, increased risk behaviors, and lower engagement with HIV prevention. An integrated health system with collective efforts and support among stakeholders, policymakers, local communities, and organizations working in HIV and mental health care is essential for delivering quality services and care to people who are vulnerable to HIV and people with mental health conditions, particularly during a pandemic [38]. An integrated health system is essential to achieving HHS’s *Ending the HIV Epidemic in the U.S.* initiative goal of reducing new HIV infections in the United States by 90% by 2030 [39]. Estimating the utilization of health care services and understanding the factors influencing use among MBs with HIV and depression can help predict the use of these services in different populations and provide efficient and high quality services with limited resources.

Our findings are subject to several limitations. First, our data are claims-based, and the population is restricted to MBs with FFS claims covered by Medicare insurance; claims were submitted by physicians’ offices, inpatient hospitals, or laboratories. Only patients who had claims for medical services or filled at least one prescription were included in the analysis. Our estimates cannot account for claims not submitted from health care settings, denied claims, those not accepted for treatment, or those accepted for treatment but who failed to fill their first prescription [40]. Moreover, because our analysis included data from the monthly Medicare FFS claims but did not include Medicare Advantage encounter data, our service utilization estimates are underestimated. Additionally, our estimates are likely underestimated because of delays in Medicare data collection and reporting. Moreover, demographic distributions and disease prevalence among the Medicare population are different from the U.S. general population, which includes people who are ineligible to receive Medicare services. Therefore, our estimates might not be directly comparable with those in prior studies that used data collected from the U.S. general population. Lastly, we assessed service utilization among MBs with HIV and depression by using limited information (e.g., basic demographic characteristics) available in Medicare claims data. Although we included health care services for selected life-threatening illness (e.g., HIV, depression, disability, and other chronic conditions), we did not consider the causes of depression (e.g., opioid crisis and increasing availability of guns in homes) or the subsequent outcomes of severe and untreated depression (e.g., suicide attempts, completed suicides) that might require emergency care, hospitalization, or treatment. Moreover, our study did not include other factors that might affect the use of health care services (e.g., socioeconomic disadvantage, cultural and lifestyle factors).

## 5. Conclusions

We observed that a small percentage of MBs had FFS claims for both HIV and depression in 2020 and that claims for health care services utilization were significantly associated with claims for HIV and depression. The characteristics of MBs with claims for HIV and depression were significantly different from MBs without claims for HIV or depression. MBs with claims for both HIV and depression were more likely than those without HIV or depression to have short-stay or long-stay hospitalizations, longer hospital stays, require outpatient diagnostic services, be treated with prescription drugs, and require outpatient procedures, supplies, and products.

Our findings indicate racial and ethnic inequalities in the use of health services among MBs with both HIV and depression, with slight variations by different type of health services. Non-White beneficiaries were more likely than White beneficiaries to be hospitalized but were less likely to receive drug treatment, outpatient diagnostic services, or outpatient procedures, supplies, and products during the pandemic. The reduction or elimination of racial/ethnic disparities has long been a U.S. policy goal. These findings identify and quantify factors affecting the use of health services among MBs with HIV and depression. Furthermore, policymakers, stakeholders, and public health communities can use these results and supplement with other resources to help allocate resources to develop and evaluate program-specific HIV and depression intervention, disease prevention, and needed health care services and research for the communities with different demographic distributions. This information can also be used to estimate disparity burdens on HIV- and depression-related health services and interventions for underserved populations during a public health emergency.

## Figures and Tables

**Table 1:** Characteristics of Medicare beneficiaries with fee-for-service claims for HIV and depression, United States, 2020.

Characteristics ^a^	HIV and Depression	HIV Only	Depression Only	Neither HIV nor Depression	Total
Total n (%)	46,523 (0.11)	74,389 (0.18)	6,542,197 (16.18)	33.8 M (83.53)	40.4 M (100)
Demographic and Social					
Age group, yrs					
<65	68.59	54.64	23.29	10.95	13.09
65–74	25.31	36.49	37.63	53.19	50.61
75–84	5.11	7.77	24.84	24.70	24.67
≥85	0.99	1.10	14.24	11.17	11.64
Mean	58.09	60.91	70.64	71.88	71.64
Sex					
Male	70.94	76.81	33.57	49.81	47.25
Female	29.06	23.19	66.43	50.19	52.75
Race/ethnicity					
Non-Hispanic White	49.52	40.67	82.38	75.87	76.83
Black (or African American)	34.87	42.95	7.59	9.31	9.12
Hispanic	12.24	12.10	5.78	7.40	7.15
Asian/Pacific Islander	0.90	1.32	1.57	3.66	3.31
American Indian/Alaska Native	0.62	0.63	0.61	0.53	0.54
Others/Unknown	1.85	2.32	2.08	3.24	3.05
Medicare status code ^b^					
Aged without ESRD	27.00	41.20	67.85	83.59	80.91
Aged with ESRD	1.12	1.23	0.74	0.37	0.44
Disabled without ESRD	61.27	47.53	21.50	9.93	11.92
Disabled with ESRD	2.78	3.22	0.59	0.28	0.34
ESRD only	0.69	1.18	0.18	0.22	0.21
Other	7.14	5.65	9.14	5.61	6.18
Geographic					
U.S. Census region					
Northeast	22.95	22.31	18.47	17.85	17.96
Midwest	14.19	12.30	23.41	21.31	21.62
South	43.03	46.21	40.03	37.59	38.01
West	19.64	18.71	17.95	21.12	20.60
U.S. territories	0.19	0.46	0.14	2.13	1.80
Metropolitan areas					
Metropolitan	91.07	90.68	79.28	80.22	80.09
Nonmetropolitan	8.93	9.32	20.72	19.78	19.91
Clinical					
Comorbidities					
0	4.26	14.35	3.97	36.84	31.45
1–3	42.64	54.79	43.38	42.07	42.30
4–5	26.56	19.71	28.06	14.58	16.79
≥6	26.54	11,15	25.59	6.51	9.46
End-stage renal disease					
Yes	5.58	6.25	1.96	1.03	1.20
No	94.42	93.75	98.04	98.97	98.80
Hospital outpatient emergency room visits					
≥1	53.32	33.69	46.03	18.14	22.72
0	46.68	66.31	53.97	81.86	77.28
Hospitalizations					
Short-stay hospitalization ^c^					
Yes (≥1)	34.51	18.06	30.04	10.75	13.91
No (0)	65.49	81.94	69.96	89.25	86.09
Long-stay hospitalization ^d^					
Yes (≥1)	5.29	0.93	4.04	0.76	1.30
No (0)	94.71	99.07	95.96	99.24	98.70
Average length of stay ^e^					
0 days	64.08	81.70	68.76	88.98	85.67
1 day	2.96	2.27	3.08	1.66	1.89
2–5 days	15.78	8.64	14.85	5.53	7.05
6–10 days	8.82	4.05	7.43	2.21	3.07
≥11 days	8.36	3.33	5.88	1.62	2.32
Outpatient diagnostic services					
Yes (≥1)	98.12	96.09	98.19	74.73	78.59
No (0)	1.88	3.91	1.81	25.27	21.41
Drug treatment services					
Yes (≥1)	90.95	85.57	77.28	53.10	57.11
No (0)	9.05	14.43	22.72	46.90	42.89
Outpatient procedure services					
Yes (≥1)	98.12	96.09	98.19	74.73	78.59
No (0)	1.88	3.91	1.81	25.27	21.41

ESRD: end-stage renal disease. ^a^ The characteristics of MBs with fee-for-service claims for both HIV and depression were significantly different from MBs without claims for HIV or depression by chi-squared test at *p* < 0.05. ^b^ Indicates how a beneficiary currently qualifies for Medicare in December. Other indicates beneficiary not enrolled in Medicare A or B in December. ^c^ Includes short-stay hospitalization as classified in MedPAR data (https://www.resdac.org/cms-data/variables/MEDPAR-Short-StayLong-StaySNF-Indicator-Code, accessed on 11 April 2023) based on provider number (https://resdac.org/cms-data/variables/provider-number, accessed on 11 April 2023). MedPAR provider special unit codes of “U” for swing-bed short-term/acute care hospital and “Z” for swing-bed critical access hospitals were also included. ^d^ Excludes skilled nursing facilities and short-stay hospitalization in MedPAR data (https://www.resdac.org/cms-data/variables/MEDPAR-Short-StayLong-StaySNF-Indicator-Code, accessed on 11 April 2023). ^e^ Includes short-stay and long-stay hospitalizations only. Stays in skilled nursing facilities were excluded.

**Table 2:** Health care services utilization among Medicare beneficiaries with fee-for-service claims for HIV and depression, United States, 2020.

Characteristics	Hospitalization (Short-Stay) ^b^	Hospitalization (Long-Stay) ^c,d^	Outpatient Diagnostic Services ^d^	Drug Treatment Services ^d^	Outpatient Procedure Services ^d^
	n (%)	n (%)	n (%)	n (%)	n (%)
Demographic					
Age group, yrs					
<65	9597 (32.88)	1698 (5.82)	28,628 (98.09)	27,076 (92.78)	28,628 (68.57)
65–74	3853 (35.78)	431 (4.00)	10,581 (98.25)	9439 (87.64)	10,581 (98.25)
75–84	983 (45.22)	94 (4.32)	2127 (97.84)	1839 (84.59)	2126 (97.79)
≥85	251 (59.34)	NR	417 (98.58)	348 (82.27)	417 (98.58)
Sex					
Male	10,098 (33.45)	1574 (5.21)	29,565 (97.94)	27,182 (90.05)	29,565 (97.94)
Female	4586 (37.09)	675 (5.46)	12,188 (98.57)	11,520 (93.17)	12,187 (98.56)
Race/ethnicity					
Non-Hispanic White	6503 (30.86)	1075 (5.10)	20,716 (98.32)	18,877 (89.59)	20,716 (98.32)
Black (or African American)	6031 (40.65)	892 (6.01)	14,529 (97.92)	13,720 (92.47)	14,529 (97.92)
Hispanic	1648 (31.64)	205 (3.94)	5097 (97.87)	4837 (92.88)	5096 (97.85)
Asian/Pacific Islander	113 (29.50)	NR	377 (98.43)	359 (93.73)	377 (98.43)
American Indian/Alaska Native	107 (40.38)	NR	265 (100.0)	238 (89.81)	265 (100.0)
Others/Unknown	282 (35.88)	45 (5.73)	769 (97.84)	671 (85.37)	769 (97.84)
Medicare status code ^a^					
Aged without ESRD	3696 (32.17)	404 (3.52)	11,327 (98.60)	10,050 (87.48)	11,326 (98.59)
Aged with ESRD	360 (75.47)	NR	471 (98.74)	425 (89.10)	471 (98.74)
Disabled without ESRD	7381 (28.31)	1460 (5.60)	25,650 (98.39)	24,316 (93.28)	25,650 (98.39)
Disabled with ESRD	873 (73.67)	66 (5.57)	1181 (99.66)	1106 (93.33)	1181 (99.66)
ESRD only	214 (73.04)	NR	289 (98.63)	251 (85.67)	289 (98.63)
Other	2160 (71.10)	273 (8.99)	2834 (93.29)	2554 (84.07)	2834 (93.29)
Geographic					
U.S. Census region					
Northeast	3442 (35.25)	312 (3.20)	9554 (97.85)	9056 (92.75)	9553 (97.84)
Midwest	2112 (34.97)	302 (5.00)	5943 (98.41)	5577 (92.35)	5943 (98.41)
South	6612 (36.11)	1256 (6.86)	17,959 (98.08)	16,400 (89.57)	17,959 (98.08)
West	2486 (29.75)	366 (4.38)	8239 (98.60)	7651 (91.56)	8239 (98.60)
U.S. territories	31 (38.75)	NR	56 (70.00)	NR	56 (70.0)
Metropolitan areas					
Metropolitan	13,449 (34.79)	1990 (5.15)	37,941 (98.15)	35,187 (91.03)	37,940 (98.15)
Nonmetropolitan	1188 (31.35)	244 (6.44)	3730 (98.42)	3480 (91.82)	3730 (98.42)
Clinical					
Comorbidities					
0	147 (8.11)	NR	1737 (95.86)	1628 (89.85)	1737 (95.86)
1–3	3256 (17.94)	547 (3.01)	17,720 (97.66)	16,498 (90.92)	17,720 (97.66)
4–5	3990 (35.30)	668 (5.91)	11,114 (98.34)	10,281 (90.97)	11,114 (98.34)
≥6	7291 (64.57)	1018 (9.02)	11,182 (99.03)	10,295 (91.17)	11,181 (99.02)
End-stage renal disease					
Yes	1818 (76.55)	156 (6.57)	2353 (99.07)	2135 (89.89)	2353 (99.07)
No	12,866 (32.02)	2093 (5.21)	39,400 (98.07)	36,567 (91.02)	39,399 (98.07)
Hospital outpatient emergency room visits (No. per year)					
≥1	13,646 (60.14)	2037 (8.98)	22,528 (99.29)	20,853 (91.90)	22,528 (99.29)
0	1038 (5.23)	212 (1.07)	19,225 (96.80)	17,849 (89.87)	19,224 (96.79)

ESRD: end-stage renal disease; NR: not reportable for observations < 30. ^a^ Indicates how a beneficiary currently qualifies for Medicare in December. Other indicates beneficiary not enrolled in Medicare A or B in December. ^b^ Includes short-stay hospitalization as classified in MedPAR data (https://www.resdac.org/cms-data/variables/MEDPAR-Short-StayLong-StaySNF-Indicator-Code, accessed on 11 April 2023) based on provider number (https://resdac.org/cms-data/variables/provider-number, accessed on 11 April 2023). MedPAR provider special unit codes of “U” for swing-bed short-term/acute care hospital and “Z” for swing-bed critical access hospitals were also included. ^c^ Excludes skilled nursing facilities and short-stay hospitalization in MedPAR data (https://www.resdac.org/cms-data/variables/MEDPAR-Short-StayLong-StaySNF-Indicator-Code, accessed on 11 April 2023). ^d^ No significant difference at a two-tailed chi-squared test with *p* < 0.05 by age group for outpatient diagnostic services and outpatient procedure services; by sex for long-stay hospitalizations; by metropolitan areas for outpatient diagnostic services, drug treatment services, and outpatient procedure services; by comorbidities for drug treatment services; and by end-stage renal disease for drug treatment services.

**Table 3:** Association of health care services utilization with HIV and depression fee-for-service claims among Medicare beneficiaries, United States, 2020.

Characteristics	Hospitalization (Short-Stay) ^a^	Hospitalization (Long-Stay) ^b^	Outpatient Diagnostic Services	Drug Treatment Services	Outpatient Procedure Services
	aOR (95% CI)	aOR (95% CI)	aOR (95% CI)	aOR (95% CI)	aOR (95% CI)
Demographic and social					
Age group, yrs					
≥85	1.8 (1.8–1.8) *	0.9 (0.9–0.9) *	0.8 (0.8–0.8) *	0.7 (0.7–0.7) *	0.8 (0.8–0.8) *
75–84	1.4 (1.4–1.4) *	0.8 (0.8–0.8) *	1.2 (1.2–1.2) *	0.8 (0.8–0.8) *	1.2 (1.2–1.2) *
65–74	1.1 (1.1–1.1) *	0.7 (0.7–0.7) *	0.9 (0.9–0.9) *	0.7 (0.7–0.7) *	0.9 (0.9–0.9) *
<65	Ref	Ref	Ref	Ref	Ref
Sex					
Male	1.2 (1.2–1.2) *	1.2 (1.2–1.2) *	0.6 (0.6–0.6) *	0.7 (0.7–0.7) *	0.6 (0.6–0.6) *
Female	Ref	Ref	Ref	Ref	Ref
Race/ethnicity					
Black (or African American)	1.1 (1.1–1.1) *	1.1 (1.0–1.1) *	0.5 (0.5–0.5) *	0.8 (0.8–0.8) *	0.5 (0.5–0.5) *
Hispanic	1.0 (1.0–1.0) *	1.0 (1.0–1.0) *	0.5 (0.5–0.5) *	1.0 (1.0–1.0) *	0.5 (0.5–0.5) *
Asian/Pacific Islander	0.8 (0.8–0.8) *	0.7 (0.6–0.7) *	0.6 (0.6–0.6) *	1.2 (1.2–1.2) *	0.6 (0.6–0.6) *
American Indian/Alaska Native	1.4 (1.4–1.4) *	1.4 (1.4–1.4) *	0.7 (0.7–0.7) *	0.7 (0.6–0.7) *	0.7 (0.7–0.7) *
Others/Unknown	0.9 (0.9–0.9) *	0.9 (0.9–1.0) *	1.0 (1.0–1.0) *	1.1 (1.1–1.1) *	1.0 (1.0–1.0) *
Non-Hispanic White	Ref	Ref	Ref	Ref	Ref
Geographic					
U.S. Census region					
Midwest	1.0 (0.9–1.0) *	1.4 (1.4–1.4) *	1.3 (1.3–1.3) *	0.9 (0.9–0.9) *	1.3 (1.3–1.7) *
South	0.9 (0.9–0.9) *	1.4 (1.3–1.4) *	1.2 (1.2–1.2) *	0.7 (0.7–0.7) *	1.2 (1.2–1.2) *
West	0.8 (0.8–0.8) *	1.4 (1.4–1.4) *	1.6 (1.3–2.0) *	0.8 (0.8–0.8) *	1.2 (1.2–1.2) *
U.S. territories	NR	NR	NR	NR	NR
Northeast	Ref	Ref	Ref	Ref	Ref
Metropolitan areas					
Nonmetropolitan	0.8 (0.8–0.8) *	2.3 (2.3–2.3) *	1.4 (1.4–1.4) *	1.2 (1.2– 1.2) *	1.4 (1.4–1.4) *
Metropolitan	Ref	Ref	Ref	Ref	Ref
Clinical					
Comorbidities					
≥6	4.4 (4.4–4.4) *	4.3 (4.2–4.3) *	41.6 (41.2–42.0) *	7.5 (7.5–7.5) *	41.6 (41.2–42.0) *
4–5	1.8 (1.8–1.8) *	2.5 (2.5–2.5) *	55.3 (55.0–55.7) *	7.2 (7.2–7.2) *	55.3 (55.0–55.6) *
1–3	0.9 (0.9–0.9) *	1.6 (1.5–1.6) *	43.8 (43.7–44.0) *	6.4 (6.3–6.4) *	43.8 (43.7–44.0) *
0	Ref	Ref	Ref	Ref	Ref
End-stage renal disease					
Yes	2.9 (2.9–2.9) *	0.9 (0.9–1.0) *	0.8 (0.8–0.8) *	1.1 (1.1–1.1) *	0.8 (0.8–0.8) *
No	Ref	Ref	Ref	Ref	Ref
Hospital outpatient emergency room visits (No. per year)					
≥1	16.0 (16.0–16.1) *	10.2 (10.1–10.3) *	12.5 (12.4–12.6) *	1.1 (1.1–1.1) *	12.5 (12.4–12.6) *
0	Ref	Ref	Ref	Ref	Ref
HIV and depression claims					
HIV and depression	1.6 (1.5–1.6) *	2.5 (2.4–2.6) *	4.4 (3.8–4.5) *	4.9 (4.7–5.1) *	4.2 (3.8–4.5) *
HIV only	1.0 (1.0–1.1) *	0.7 (0.7–0.8) *	7.0 (6.7–7.3) *	4.4 (4.3–4.5) *	7.0 (6.7–7.3) *
Depression only	1.5 (1.5–1.5) *	2.2 (2.1–2.2) *	2.9 (2.9–2.9) *	1.4 (1.4–1.4) *	2.9 (2.9–2.9) *
Neither HIV nor depression	Ref	Ref	Ref	Ref	Ref

aOR: adjusted odds ratio; CI: confidence interval; Ref: reference; NR: not reportable for observations < 30. ^a^ Includes short-stay hospitalization as classified in MedPAR data (https://www.resdac.org/cms-data/variables/MEDPAR-Short-StayLong-StaySNF-Indicator-Code, accessed on 11 April 2023) based on provider number (https://resdac.org/cms-data/variables/provider-number, accessed on 11 April 2023). MedPAR provider special unit codes of “U” for swing-bed short-term/acute care hospital and “Z” for swing-bed critical access hospitals were also included. ^b^ Excludes skilled nursing facilities and short-stay hospitalization in MedPAR data (https://www.resdac.org/cms-data/variables/MEDPAR-Short-StayLong-StaySNF-Indicator-Code, accessed on 11 April 2023). * Significant difference at a two-tailed chi-squared test with *p* < 0.05.

**Table 4:** Association of health care services utilization with HIV and depression fee-for-service claims among Medicare beneficiaries—by race/ethnicity ^a^, United States, 2020.

Characteristics	Non-Hispanic White	Non-Hispanic Black	Hispanic
	aOR (95% CI)	aOR (95% CI)	aOR (95% CI)
Hospitalization (short-stay) ^b^			
HIV and depression claims			
HIV and depression	1.6 (1.5–1.7) *	1.7 (1.6–1.7) *	1.5 (1.4–1.6) *
HIV only	1.0 (1.1–1.2) *	1.1 (1.0–1.1) *	1.0 (1.0–1.1)
Depression only	1.5 (1.5–1.5) *	1.5 (1.5–1.5) *	1.3 (1.3–1.4) *
Neither HIV nor depression	Ref	Ref	Ref
Hospitalization (long-stay) ^c^			
HIV and depression claims			
HIV and depression	2.6 (2.5–2.8) *	2.6 (2.4–2.8) *	2.1 (1.8–2.4) *
HIV only	0.8 (0.7–0.9) *	0.7 (0.7–0.8) *	0.7 (0.5–0.9) *
Depression only	2.1 (2.1–2.1) *	2.7 (2.6–2.7) *	2.4 (2.3–2.5) *
Neither HIV nor depression	Ref	Ref	Ref
Outpatient diagnostic services			
HIV and depression claims			
HIV and depression	4.0 (3.6–4.5) *	3.0 (2.6–3.4) *	5.3 (4.2–6.8) *
HIV only	7.0 (6.5–7.5) *	5.5 (5.2–5.9) *	7.1 (6.3–8.0) *
Depression only	2.9 (2.8–2.9) *	2.3 (2.3–2.4) *	3.4 (3.3–3.4) *
Neither HIV nor depression	Ref	Ref	Ref
Drug treatment services			
HIV and depression claims			
HIV and depression	4.2 (4.0–4.4) *	5.5 (5.2–5.9) *	6.1 (5.4–6.9) *
HIV only	3.7 (3.6–3.8) *	4.9 (4.7–5.0) *	5.9 (5.5–6.4) *
Depression only	1.4 (1.4–1.4) *	1.6 (1.6–1.7) *	1.7 (1.7–1.7) *
Neither HIV nor depression	Ref	Ref	Ref
Outpatient procedure services			
HIV and depression claims			
HIV and depression	4.0 (3.6–4.5) *	3.0 (2.6–3.4) *	5.3 (4.2–6.7) *
HIV only	7.0 (6.5–7.5) *	5.5 (5.2–5.9) *	7.1 (6.3–8.0) *
Depression only	2.9 (2.8–2.9) *	2.3 (2.3–2.4) *	3.4 (3.3–3.4) *
Neither HIV nor depression	Ref	Ref	Ref

aOR: adjusted odds ratio; CI: confidence interval; Ref: reference. ^a^ Asian/Pacific Islander and American Indian/Alaska Native MBs are excluded because of insufficient samples. ^b^ Includes short-stay hospitalization as classified in MedPAR data (https://www.resdac.org/cms-data/variables/MEDPAR-Short-StayLong-StaySNF-Indicator-Code, accessed on 11 April 2023) based on provider number (https://resdac.org/cms-data/variables/provider-number, accessed on 11 April 2023). MedPAR provider special unit codes of “U” for swing-bed short-term/acute care hospital and “Z” for swing-bed critical access hospitals were also included. ^c^ Excludes skilled nursing facilities and short-stay hospitalization in MedPAR data (https://www.resdac.org/cms-data/variables/MEDPAR-Short-StayLong-StaySNF-Indicator-Code, accessed on 11 April 2023). * Significant by chi-squared test at *p* < 0.05 and adjusted for age, sex, region, metropolitan, comorbidity, end-stage renal disease, and hospital outpatient emergency room visits.

## Data Availability

Data used in this study are available through the Centers for Medicare and Medicaid Services (CMS, https://www.cms.gov/, accessed on 11 April 2023). Data are also available through the Research Data Assistance Center (ResDAC, https://resdac.org, accessed on 11 April 2023).
